# Effects of CRISPR/Cas9-mediated stearoyl-Coenzyme A desaturase 1 knockout on mouse embryo development and lipid synthesis

**DOI:** 10.7717/peerj.13945

**Published:** 2022-09-14

**Authors:** Huibin Tian, Huimin Niu, Jun Luo, Weiwei Yao, Wenchang Gao, Yang Wen, Min Cheng, Anmin Lei, Jinlian Hua

**Affiliations:** 1 Shaanxi Key Laboratory of Molecular Biology for Agriculture, College of Animal Science and Technology, Northwest A&F University, Yangling, Shaanxi, China; 2 College of Veterinary Medicine, Shaanxi Centre of Stem Cells Engineering & Technology, Northwest A&F University, Yangling, Shaanxi, China

**Keywords:** Stearoyl-Coenzyme A desaturase 1, CRISPR/Cas9, Knockout mouse, Embryo development, Lipid synthesis

## Abstract

**Background:**

Lipid synthesis is an indispensable process during embryo and growth development. Abnormal lipid synthesis metabolism can cause multiple metabolic diseases including obesity and hyperlipidemia. Stearoyl-Coenzyme A desaturase 1 (SCD1) is responsible for catalyzing the synthesis of monounsaturated fatty acids (MUFA) and plays an essential role in lipid metabolism. The aim of our study was to evaluate the effects of SCD1 on embryo development and lipid synthesis in a knockout mice model.

**Methods:**

We used the CRISPR/Cas9 system together with microinjection for the knockout mouse model generation. Ten-week-old female C57BL/6 mice were used for zygote collection. RNase-free water was injected into mouse zygotes at different cell phases in order to select the optimal time for microinjection. Five sgRNAs were designed and *in vitro* transcription was performed to obtain sgRNAs and Cas9 mRNA. RNase-free water, NC sgRNA/Cas9 mRNA, and *Scd1* sgRNA/Cas9 mRNA were injected into zygotes to observe the morula and blastocyst formation rates. Embryos that were injected with *Scd1* sgRNA/Cas9 mRNA and developed to the two-cell stage were used for embryo transfer. Body weight, triacylglycerol (TAG), and cholesterol in *Scd1* knockout mice serum were analyzed to determine the effects of SCD1 on lipid metabolism.

**Results:**

Microinjection performed during the S phase presented with the highest zygote survival rate (*P* < 0.05). Of the five sgRNAs targeted to *Scd1*, two sgRNAs with relatively higher gene editing efficiency were used for *Scd1* knockout embryos and mice generation. Genome sequence modification was observed at *Scd1* exons in embryos, and *Scd1* knockout reduced blastocyst formation rates (*P* < 0.05). Three *Scd1* monoallelic knockout mice were obtained. In mice, the protein level of SCD1 decreased (*P* < 0.05), and the body weight and serum TAG and cholesterol contents were all reduced (*P* < 0.01).

## Introduction

The synthesis of fatty acids is of great importance during embryo and growth development, and fatty acids participate in multiple physiological metabolic processes. Disrupted lipid homeostasis can cause metabolic disorders including diabetes, hyperlipidemia, and cancer (Luo, Yang & Song, 2020; Wang et al., 2020). Monounsaturated fatty acids (MUFAs) and saturated fatty acids (SFAs) are key components of neutral lipids and essential for early embryo development and lipid synthesis (Fayezi et al., 2018; Matsuzaka et al., 2020).

The rate-limiting enzyme in the regulation of MUFA synthesis is stearoyl-CoA desaturase 1 (SCD1), which can catalyze the synthesis of palmitoleic acid and oleic acid by using palmitic acid and stearic acid as substrates (Dobrzyn & Ntambi, 2005). SCD1 is located in the endoplasmic reticulum (ER) membrane and can introduce a cis double bond formation at the cis-Δ9 position in the fatty acid chain (Bloch, 1969). Additionally, SCD1 is involved in many biological processes including adipocyte inflammation, mammary gland fatty acid synthesis, and insulin resistance (Dobrzyn, Jazurek & Dobrzyn, 2010b; Malodobra-Mazur et al., 2014; Yao et al., 2017). Inhibition of SCD1 can down-regulate expression levels of lipid droplet formation-related genes in pig embryos (Lee et al., 2019). Knockout of *Scd1* in leptin-deficient mice resulted in decreased neutral lipid content and whole-body adiposity (Dobrzyn et al., 2010a), indicating the role that SCD1 plays in lipid accumulation.

CRISPR/Cas9-mediated genome editing is a convenient and efficient tool used to induce gene modifications across a variety of cell types and animals (Shrock & Guell, 2017; Wilkinson et al., 2019). A single guide RNA (sgRNA) can direct Cas9 proteins to the targeted site, causing DNA double strands to break and inducing gene knockout or knock-in (Ran et al., 2013). With the help of microinjection technology, it is easier to obtain gene knockout embryos and transgenic animals in one-step generation (Wang et al., 2013; Wu et al., 2019). However, the process of microinjection would be harmful to zygote survival. It is important to choose a suitable cell phase when performing microinjection in order to minimize the embryo mortality rate.

Previous studies used blastocyst microinjection with embryonic stem cells to generate gene-modified mice, and this is a time-consuming process with unstable gene modification when delivered to offspring (Liégeois, Horner & De Pinho, 1996; Peli et al., 1996). The advantages of using knockout mice with CRISPR/Cas9 and zygote microinjection technology are ease, convenience, and high efficiency when compared with other methods. Moreover, CRISPR/Cas9-mediated gene knockout is more accurate in gene targeting and has a smaller off-target effect than the traditional gene target methods. Therefore, it is necessary to study the function of SCD1 in embryo development and lipid synthesis *in vivo* through CRISPR/Cas9-mediated gene editing for lipid metabolic disease treatment.

The objective of this study was to investigate the effects of SCD1 on embryo development and lipid synthesis in a knockout mouse model generated by CRISPR/Cas9 combined with microinjection. The optimal cell cycle phase for microinjection in mouse embryos was also determined.

## Materials & Methods

### Animals and ethics statement

Ten-week-old male and female C57BL/6 and ICR mice were purchased from Air Force Medical University (Shaanxi, China) and housed in a specific-pathogen-free (SPF) animal facility with a 12-h light/dark cycle at a controlled temperature (22 ± 2 °C), humidity of 40%–60%, and ad libitum access to water and food. Same sex mice were housed together in individually ventilated cages with four mice per cage. All animal protocols were performed in accordance with the approval of the Animal Ethical and Welfare Committee of College of Animal Science and Technology, Northwest A&F University, Yang Ling, China (permit number: 15-516, date: 2019-8-1).

### Isolation and cultivation of mouse zygotes

Female mice were selected randomly for zygote isolation. Superovulation was induced in female mice via intraperitoneal injection of 10 IU of pregnant mare serum gonadotropin (PMSG, Ningbo, China). After 46–48 h, 10 IU of human chorionic gonadotropin (hCG, Ningbo, China) was intraperitoneally injected in the mice. Mice were included in the study if they were in good health and in oestrus. Mice were excluded if they were ill or did not enter oestrus. There were 10 female mice treated for superovulation in the one experiment. The total number of animals used in the whole experiment was 200. Twelve hours later, female mice that had successfully mated with male mice were checked for the presence of a vaginal plug. If there was no vaginal plug, the mouse was not used in the experiment. Females were sacrificed by cervical dislocation; in detail, the mouse was removed from its cage and placed on a smooth, hard surface without releasing the tail. A metal rod was placed firmly behind the ears and across the neck, and the tail was pulled sharply to the rear while pressing down on the neck. Zygote-cumulus complexes were isolated from the most swollen oviduct in M2 medium (M7167, Sigma, St. Louis, MO, USA). The average number of zygotes isolated from each mouse was about 15, and the total number of zygotes was about 150 in one experiment. The zygotes were randomly allocated into different groups for microinjection, placed in hyaluronic acid in order to release granulosa cells, and cultured in M2 medium until microinjection. Injected zygotes were cultured in EmbryoMax^®^ KSOM Mouse Embryo Media (MR-121-D, Sigma) until the morula and blastocyst stages.

### Specific single guide RNA design and vector construction

**Figure 1:**
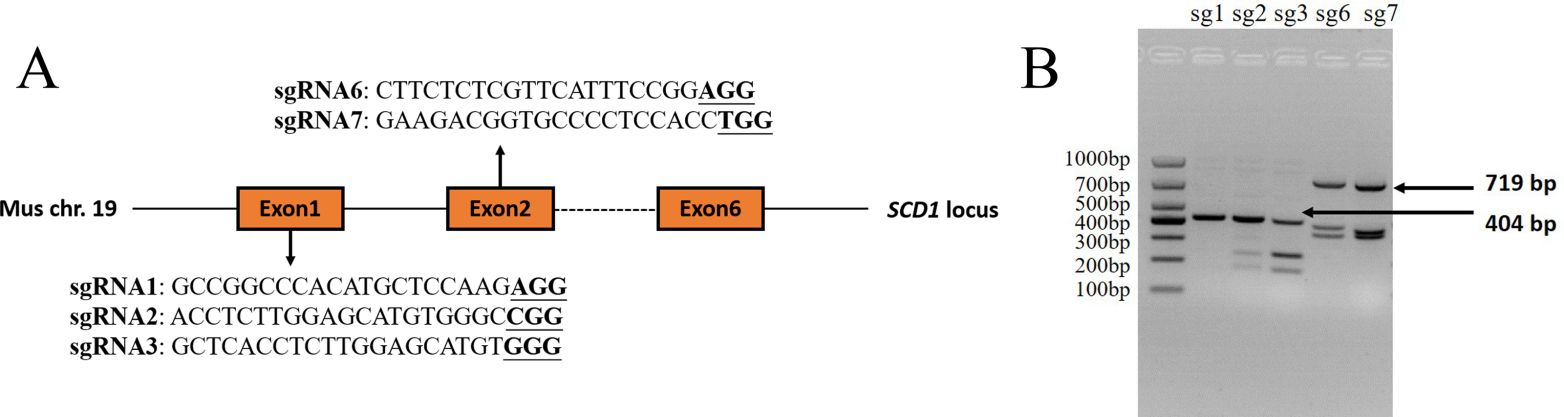
*Scd1* gene sgRNA targeted sites and sgRNA *in vitro* activity analysis. (A) *Scd1* gene locus in the *Mus musculus* genome and five sgRNAs sequenced together with PAM motif. (B) *In vitro* sgRNA activity analysis of *Scd1* five sgRNAs. sgRNA2, 3, 6, and 7 showed three bands in the gel, meaning that they had cleavage activity. The PCR product of exon 1 containing sg1, sg2, and sg3 target sites was 404 bp, and exon 2 containing sg6 and sg7 targeted sites was 719 bp. sg1, 2, 3, 6, and 7 represented the sgRNA1, sgRNA2, sgRNA3, sgRNA6, and sgRNA7 groups, respectively.

The sgRNAs that were targeted to exon 1 and exon 2 of the *Mus musculus Scd1* gene (Gene ID: 20249) were designed using the online website CHOPCHOP (http://chopchop.cbu.uib.no/) and Guide Design Resources (https://zlab.bio/guide-design-resources). Three sgRNAs that were targeted to exon 1 and two sgRNAs targeted to exon 2 were selected according to their predicted scores. The sequences of sgRNAs and PAM motifs are shown in Fig. 1A. The sgRNAs were added to the *Bsa* I (R3733S, NEB, Ipswich, MA, USA) restriction enzyme site according to a previous study (Shen et al., 2013) and synthesized as single-strand DNA oligonucleotides by Sangon Biotech (Shanghai, China). The oligonucleotides were then annealed and inserted into a pUC57-sgRNA expression vector using T4 DNA ligase (2011A, Takara Bio Inc., Otsu, Japan) according to the manufacturer’s instructions. The negative control sgRNA (NC sgRNA) sequence (5′-ACCGGAAGAGCGACCTCTTCT-3′), which did not target to any sites in the mouse genome according to a previous report (Yang et al., 2017), was also synthesized and constructed into the vector, which was used as a control in the experiment.

### *In vitro* transcription of sgRNA, Cas9 mRNA, and analysis of sgRNA activity

pST1374-NLS-flag-linker-Cas9 vector (Addgene#44758) was linearized with *Age* I (R3552S, NEB) and used for Cas9 mRNA *in vitro* transcription using the T7 Ultra Kit (AM1345; Invitrogen, Waltham, MA, USA), and the Cas9 mRNA was purified using an RNeasy Mini Kit (74104; Qiagen, Hilden, Germany). For sgRNA *in vitro* transcription, the DNA templates were obtained using PCR amplification from pUC57-sgRNA expression vectors according to a previous study (Shen et al., 2014). In brief, the PCR was amplified using PrimeSTAR Max DNA Polymerase (R045A; Takara, Shiga, Japan) according to the manufacturer’s protocol and with the following primers: forward primer, 5′- TCTCGCGCGTTTCGGTGATGACGG-3′and reverse primer, 5′- AAAAAAAGCACCGACTCGGTGCCACTTTTTC-3′. The PCR conditions were as follows: 98 °C for 10 s, followed by 56 °C for 5 s, and 72 °C for 5 s, 33 cycles in total. The PCR product was run on 1.5% agarose gels and purified using an AxyPrep DNA Gel Extraction Kit (AP-GX-50, Axygen, NY, USA). sgRNA *in vitro* transcription was performed using a MEGAshortscript™ Kit (AM1354, Invitrogen, Waltham, MA, USA) and purified using a MEGAclear™ Kit (AM1908, Invitrogen, Waltham, MA, USA).

To detect the activity of sgRNAs obtained by *in vitro* transcription, *Scd1* gene exon 1 and exon 2 fragments containing sgRNA target sites were obtained by PCR and used for sgRNA activity experiments. The primers used for exon 1 (E1) and exon 2 (E2) amplification were as follows: E1-F, 5′-AATACTGAACACGGTCATCCCA-3′; E1-R, 5′- ACTCATCTGCCCAAATTACAATC-3′; E2-F, 5′- ATCTGTTTTCCGATGGTCTT-3′; E2-R, 5′- ACAGGGACTCAGTATTCATGTTA-3′. PCR amplification was performed under the reaction conditions: 98 °C for 10 s, 55 °C for 5 s, and 72 °C for 10 s. Individual sgRNA at 100 ng and targeted DNA fragments at 300 ng were mixed with 0.3 µL Cas9 Nuclease, S. pyogenes (M0646M; NEB, Ipswich, MA, USA), and 10 × NEBuffer 3.1 at 20 µL volume in total. The mixture was then incubated at 37 °C for 30 min. The DNA fragments were purified using AxyPrep PCR Cleanup Kit (AP-PCR-50, Axygen) and agarose gel electrophoresis was performed to analyze whether the DNA fragments were cut off by sgRNA and Cas9 complex in order to examine sgRNA activity *in vitro*.

### sgRNA/Cas9 cytoplasmic microinjection

In order to determine the optimal cell cycle phase for microinjection, RNase-free water was injected into the cytoplasm of mouse zygotes at the G1, S, and G2 phases using the FemtoJect system (Eppendorf, Hamburg, Germany) at 400 hPa injection pressure, 30 hPa compensatory pressure, and 0.3 s injection time. The G1, S, and G2 phases were determined to be 8 to 10 h, 14 to 16 h, and 18 to 20 h postfertilization, respectively, according to a previous study (Gu, Posfai & Rossant, 2018). The embryo survival rates were calculated immediately after microinjection. In order to select sgRNAs with higher gene-editing efficiency, each sgRNA (10 ng/µL) was separately mixed with Cas9 mRNA (20 ng/µL), the mixture was injected into the zygotes, and then the zygotes were cultured in KSOM at 37 °C with 5% CO_2_ until morulae or blastocysts formed for genotyping. In order to explore the effects of *Scd1* knockout on embryo development, zygotes were divided into three groups for microinjection at the optimal cell cycle phase: RNase-free water, NC sgRNA with Cas9 mRNA, and *Scd1* sgRNA with Cas9 mRNA. The morulae and blastocysts were observed 3.5 days following fertilization and the rates of morula and blastocyst formation were calculated.

### Mouse embryo transfer

The embryo transfer was conducted as previously described (Kolbe et al., 2012). The pseudo-pregnant foster mothers were prepared by mating ten-week-old ICR female mice with vasectomized male mice on the same day the zygote donor mice were mated. Successful mating was determined by the presence of a vaginal plug the following morning. Before embryo transfer, the surrogate mothers were checked again to determine whether ovulation had occurred. Females with a swollen ampulla (the sign of ovulation) were used as surrogate mothers. After zygotes were injected with Cas9 mRNA and sgRNA mixture for 24 h, the two-cell stage embryos were transferred into pseudo-pregnant foster mouse oviducts. Females were anesthetized via intraperitoneal injection and placed on a warm plate at 37 °C. The mouse’s back area near the ovary was shaved and disinfected with ethanol. An incision was made on the right side to pull out the ipsilateral ovary, oviduct, and uterine horn. The thin transparent connective tissue membrane was opened, and 16 two-cell embryos were transferred into the ampulla. The reproductive tract was then placed back inside and the incision was sutured. There were no adverse events during embryo transfer. The pups were delivered about 21 days after embryo transfer. The mice were weighed every week after birth. The surviving animals were kept alive at the conclusion of the experiment.

### Genotyping of mouse embryos and tails

The single embryos that developed to morulas or blastocysts were collected for genomic DNA extraction using a REPLI-g Single Cell Kit (150343, Qiagen, Hilden, Germany). PCR was performed to amplify the exon 1 and exon 2 of the *Scd1* gene using PrimeSTAR Max DNA Polymerase (R045A, Takara, Shiga, Japan) with the reaction conditions as follows: 98 °C for 10 s, 55 °C for 5 s, and 72 °C for 5 s. The primers were the same as those used for the sgRNA *in vitro* activity analysis experiment. The PCR product was used for Sanger sequencing and inserted into the pMD19-T vector to verify the sequence mutation in the genome.

Mice tails were isolated by biopsy three weeks after birth. Mouse tail genomic DNA was extracted using a Universal Genomic DNA Kit (CW2298S, CW Biotech, Beijing, China). The PCR products of exon 1 and exon 2 were obtained using the same method as that of the embryos and purified using a PCR Clean-Up Kit (AP-PCR-50, Axygen, NY, USA). Purified DNA was annealed for T7EN1 cleavage assay (M0302L, NEB, Ipswich, MA, USA) and analyzed using agarose gel electrophoresis according to a previous study (Vouillot, Thelie & Pollet, 2015). The DNA was then inserted into a pMD19-T vector. A single bacterial colony was picked up for sequencing by Sangon Biotech (Shanghai, China) to evaluate the nucleotide modifications.

### Protein isolation and Western blot

The mice born after embryo transfer that were identified as wild type were used as the control group to be compared with *Scd1* gene modified mice in the following experiments. For each animal, three different investigators were involved in the experiment as follows: the first investigator collected the samples and was the only person aware of the group allocation. A second investigator was responsible for the sample analysis. Finally, a third investigator (also unaware of the mouse’s group) assessed the data analysis.

Mouse tail tissue was placed in liquid nitrogen to be ground and then lysed in ice-cold RIPA buffer (R0010; Solarbio, Beijing, China) with protease inhibitor (04693132001; Roche Diagnostics Ltd., Mannheim, Germany). A BCA protein assay kit (23227; Thermo Fisher Scientific, Rockford, IL, USA) was used for protein concentration measurement. Protein was separated using 10% SDS-PAGE and transferred onto a nitrocellulose membrane (HATF00010; Millipore, Burlington, MA, USA). After being blocked for 1.5 h using 5% skim milk, the membranes were probed with primary antibodies, monoclonal mouse anti-SCD1 (ab19862, 1:1000; Abcam, Cambridge, MA, USA), and monoclonal mouse anti- *β*-tubulin (CW0098, 1:1000; CW Biotech, Beijing, China). The membranes were washed three times with TBST, and HRP-conjugated goat anti-mouse-IgG (CW0102, 1:2000; CW Biotech) was used as a secondary antibody. Signals were detected using an ECL Western blot system (1705061, Bio-Rad, Hercules, CA, USA). ImageJ software was used to analyze the densities of the bands and SCD1 relative expression was normalized to *β*-tubulin.

### Measurement of total serum triacylglycerol (TAG) and cholesterol

Total serum TAG and cholesterol were detected using a GPO-Trinder TAG assay kit (E1003; Applypen Technologies Inc., Beijing, China) and a cholesterol assay kit (E1005; Applypen Technologies Inc.). The tip of the mouse tail was clipped, and blood was collected from the tail using a hand-held pipette when the mice reached four and eight weeks old. Serum was collected from clotted blood and used for TAG and cholesterol assays at 550 nm on a Biotek microplate reader (Winooski, VT, USA).

### Statistical analysis

All data were analyzed using SPSS 19.0 (SPSS, Inc., Chicago, IL, USA) and the results were presented as means ±SEM with a 95% confidence interval. Significance across the three groups was analyzed using one-way ANOVA and a general linear model procedure, and lowercases (a to c) denoted significant differences (*P* < 0.05). The statistical analysis of the other parameters between the two groups was performed using the Student’s *t*-test and statistical significance was considered at *P < 0.05 and **P < 0.01.

## Results

### Zygote microinjection during the S phase showed the highest embryonic survival rate

The mouse zygotes that were injected with RNase-free water at different cell cycle phases and microinjected during the S phase had the highest embryonic survival rate (*P* < 0.05) compared with those injected during the G1 and G2 phases (Table 1). This result indicated that the S phase was the optimal time for microinjection with less damage to zygotes.

### Four sgRNAs targeted to *Scd1* revealed *in vitro* activity

Of the five sgRNAs (Fig. 1A), sgRNA2 and sgRNA3 targeted to exon 1 of the *Scd1* gene, and sgRNA6 and sgRNA7 targeted to exon 2, showed *in vitro* activity (Fig. 1B). Based on these results, sgRNA2, 3, 6, and 7 were used for *Scd1* knockout in mouse zygotes.

### Gene editing efficiency of different sgRNAs and *Scd1* gene sequence modification in mouse embryos

**Figure 2:**
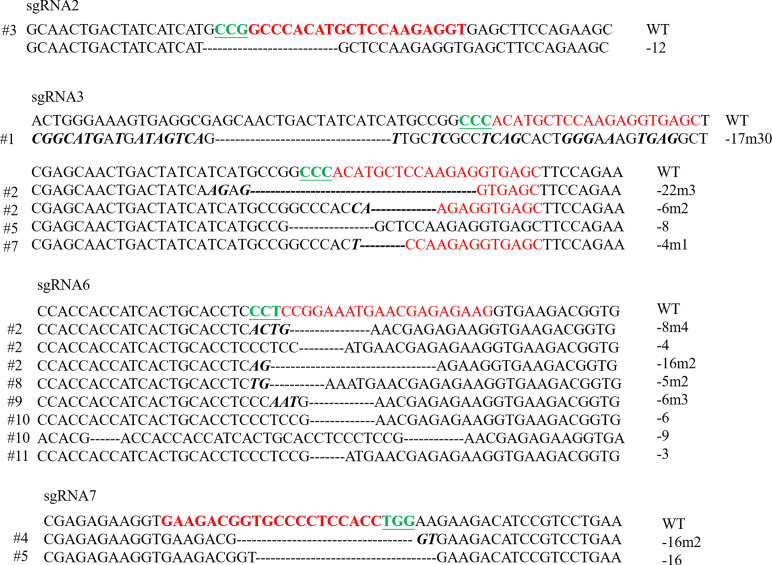
Sequences of modified *Scd1* alleles in each embryo for four sgRNAs. sgRNA target sites are in red; PAM motifs are green and underlined; mutations are bold and italic; deletion (-), mutation (m) and wild type (WT) are shown at the right.

Individual sgRNA was mixed with Cas9 mRNA, and four groups of RNA mixture were used for cytoplasmic microinjection in the S phase. The gene editing efficiencies of sgRNA2, 3, 6, and 7 were 9.1%, 36.4%, 45.6%, and 20.0%, respectively (Table 2). DNA sequences at the sgRNA targeted sites were also analyzed. One embryo had two genotypes in the sgRNA3 group, and the sgRNA6 group had one embryo with two genotypes and one embryo with three genotypes, indicating that these embryos were mosaicisms (Fig. 2).

### Knockout of *Scd1* prevented blastocyst formation

**Figure 3:**
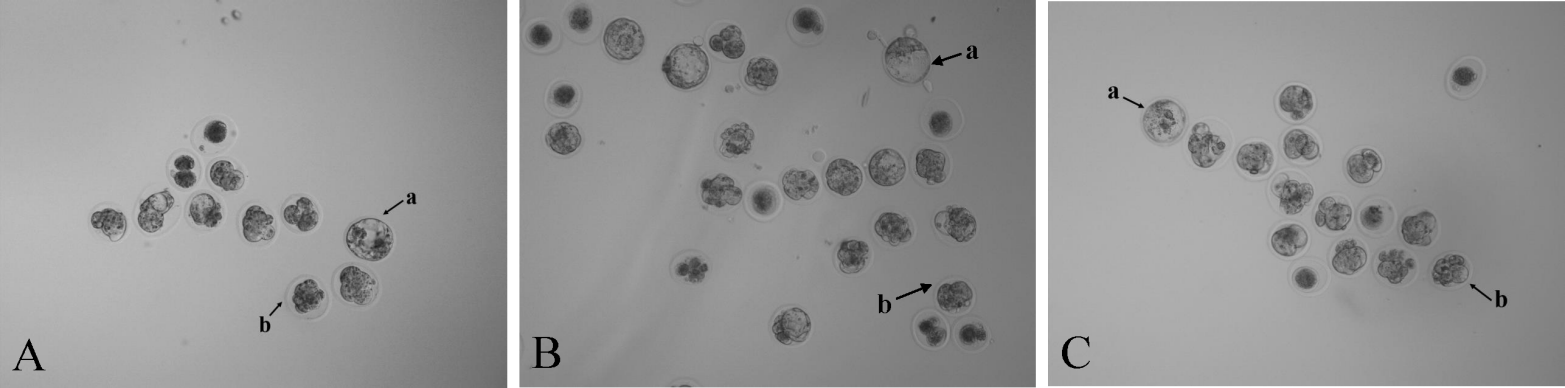
Photomicrographs of blastocysts and morulae in three treatment groups (40 ×). Mouse embryos microinjected with (A) RNase-free water, (B) NC sgRNA/Cas9 mRNA, and (C) *Scd1* sgRNA/Cas9 mRNA. The letter a indicates the blastocyst and letter b indicates the morula.

Zygotes injected with NC sgRNA and Cas9 mRNA had a lower blastocyst formation rate (*P* < 0.05) compared with the RNase-free water group. The embryo images of the three groups’ morulae and blastocysts are shown in Fig. 3. Compared with the two control groups, the blastocyst formation rate decreased (*P* < 0.05) after injection with *Scd1* sgRNA and Cas9 mRNA (Table 3). This indicated that knockout of *Scd1* prevented zygote development into blastocyst, and microinjection of sgRNA and Cas9 mRNA had a negative effect on embryo development.

### Sequence modifications at *Scd1* gene locus in mice

**Figure 4:**
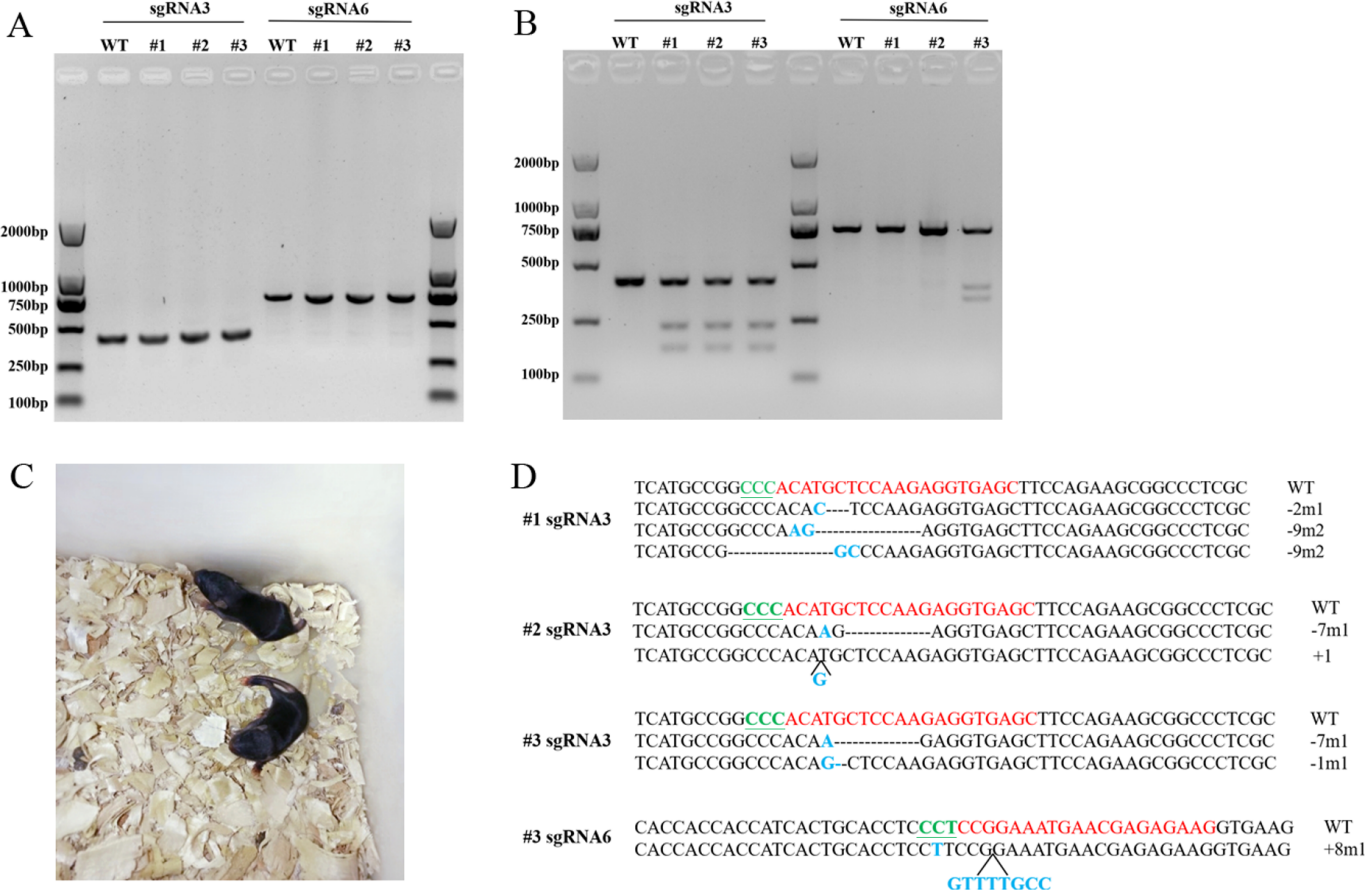
T7EN1 assay and sequence analysis of sgRNA3 and sgRNA6 target sites of mice. (A) PCR products of sgRNA3 and sgRNA6 target sites amplified from mice genomic DNA. The lengths of the PCR fragments including sgRNA3 and sgRNA6 target sites were 404 bp and 719 bp, respectively. (B) T7EN1 assay to detect sequence modifications of two sgRNA target sites. (C) The image of *Scd1* knockout mice. (D) The genome sequences of three mice. sgRNA target site sequences are in red; PAM motifs are in green and underlined; mutations and insertions are in blue; deletion (-), mutation (m), insertion (+) and wild type (WT) are shown at the right. #1, #2, and #3 represents the three mice.

We used sgRNA3 targeted to exon 1 and sgRNA6 targeted to exon 2, which had relatively higher gene editing efficiency, for *Scd1* knockout mice generation. Six mice were born after embryo transfer, and PCR was performed to obtain the fragments of exon 1 and exon 2, as shown in Fig. 4A. Of these mice, three were identified with *Scd1* sequence modifications at sgRNA target sites by T7EN1 assay and sequencing (Figs. 4B and 4D). The photograph of the knockout mice is shown in Fig. 4C. Two mice had nucleotide mutations only in sgRNA3 target sites, and another mouse genome had sequence modifications at both sgRNA3 and sgRNA6 target sites (Fig. 4D). The wild type genotype could be detected in three mice with mutations, suggesting that they were *Scd1* monoallelic knockout mice. Three wild type mice that were born after embryo transfer were used as the control group in the following experiment.

**Table 1:** Effect of microinjection in different cell cycle phases on mouse embryonic survival rate. The survival rates were calculated immediately after microinjection as the ratio of the number of survival embryos to total injected embryos. Data are shown as means ± SEM for at least three biological repeats. The different letters (a, b, c) mean significant differences (*P* < 0.05) in different groups.

**Cell cycle phase for injection**	**No. of injected embryos**	**No. of survival embryos**	**Survival rate (%)**
G1 phase	159	55	34.96^c^± 2.13
S phase	130	124	94.90^a^± 4.11
G2 phase	139	122	89.14^b^± 7.62

**Table 2:** Gene editing efficiency of *Scd1* gene different sgRNAs in mouse embryos.

**sgRNA**	**No. of embryos containing sequence modifications**	**Total no. of embryos**	**Gene editing efficiency (%)**
sgRNA2	1	11	9.1
sgRNA3	4	11	36.4
sgRNA6	5	11	45.6
sgRNA7	2	10	20.0

**Notes.**

The different sgRNAs gene editing efficiency were calculated as ratio of embryos with sequence modifications and total number of embryos detected.

### *Scd1* monoallelic knockout mice showed decreased body weights, serum TAG, and cholesterol

**Figure 5:**
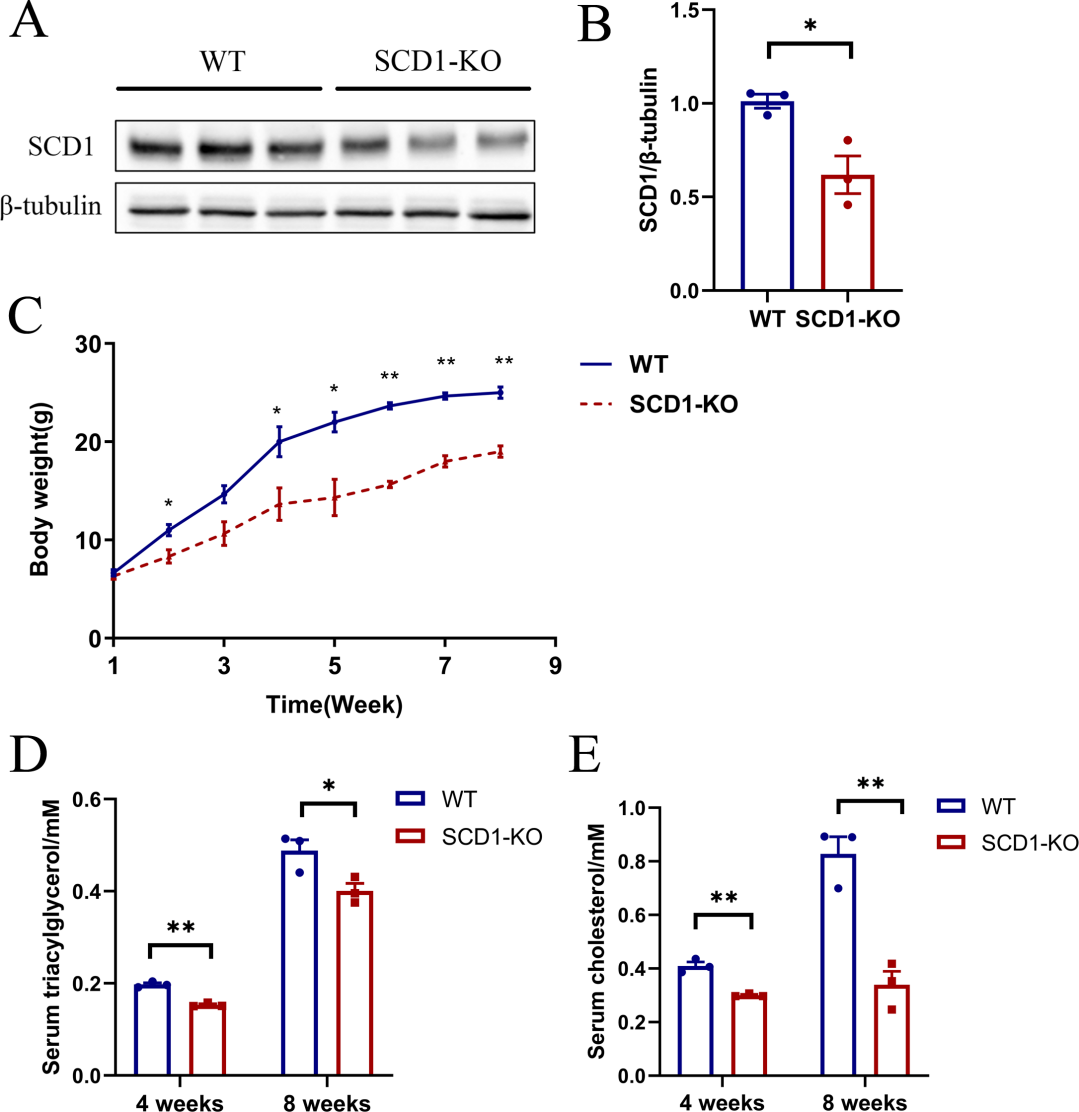
The SCD1 protein level, body weight, serum TAG, and cholesterol contents of *Scd1* knockout mice. (A) SCD1 protein level in wild type and knockout mice. (B) Body weight changes of mice from one to eight weeks. (C) TAG and (D) cholesterol contents in mice serum. SCD1-KO, SCD1 knockout; WT, wild type; data are presented as means ±SEM, *n* = 3 for each group. ^∗^*P* < 0.05 vs. control; ^∗∗^*P* < 0.01 vs control.

The *Scd1* knockout mice were inactive compared with the wild type mice. However, it took a longer time for the knockout mice to be mated. *Scd1* knockout (*n* = 3) and wild type mice (*n* = 3) were analyzed for protein, serum TAG, and cholesterol detection. The SCD1 protein level decreased (*P* <  0.05) by 40% in *Scd1* sequence modified mice (Figs. 5A and 5B), which also indicated monoallelic knockout of *Scd1* in mice. The body weights of *Scd1* knockout mice showed a continuous decrease (*P* < 0.05) from four weeks compared with the wild type mice (Fig. 5C). Additionally, the serum TAG and cholesterol reduced in *Scd1* knockout mice (*P* < 0.01) (Figs. 5D and 5E).

## Discussion

Many metabolic diseases are caused by the overaccumulation of lipids. Understanding the role of lipid synthesis regulation factors in the mouse model will contribute to the treatment of lipid metabolic disorders and diseases. In this study, CRISPR/Cas9 technology combined with microinjection was used for *Scd1* knockout embryos and mice generation. Knockout of *Scd1* decreased the blastocyst formation rate in embryos, and body weight, serum TAG, and cholesterol were reduced in *Scd1* knockout mice. These results demonstrated that SCD1 was an important regulator during embryo development and lipid synthesis, and could be used as a therapeutic target in lipid disorder treatment.

**Table 3:** Morula and blastocyst formation rate after *Scd1* knockout. The ratio of morula and blastocyst were calculated as number of morulae or blastocysts to total number of zygotes cultured in each treatment. Data are shown as means ± SEM for three biological repeats. The different letters (a, b, c) mean significant differences (*P* < 0.05) in different groups.

**Treatment**	**Total no. of zygotes**	**No. of morulae**	**Ratio of morula (%)**	**No. of blastocysts**	**Ratio of blastocyst (%)**
RNase-free water	60	16	26.73^c^±0.75	29	47.48^a^±12.10
NC sgRNA/Cas9 mRNA	73	24	33.16^b^ ±5.51	23	31.72^b^ ±1.36
*Scd1* sgRNA/Cas9 mRNA	95	37	39.46^a^± 1.87	21	21.51^c^± 11.41

Using CRISPR/Cas9 technology combined with zygote microinjection is a high-efficiency method for gene-editing mice production (Hall et al., 2018). However, microinjection causes damage to the zygotes because it disorganizes the cellular internal structures (Liu et al., 2015). The cytoskeleton changes during cell cycle transition, and microinjection during different cell phases can influence the cytoskeleton and membrane structure (Pegoraro, Janmey & Weitz, 2017), resulting in a decreased embryo survival rate and gene editing efficiency. Previous research in bovines found that microinjection in the middle of the zygotic S phase resulted in a higher embryonic development rate (Gagné, Pothier & Sirard, 1995). A recent study also reported an efficient approach to S-phase injection for large DNA donor insertion in mouse zygotes (Abe et al., 2020), which supported our findings that microinjection performed in the S phase had less damage to zygotes compared with injection in the G1 and G2 phases. The mechanism of the S phase can resist external disturbance when induced by microinjection still needs further exploration.

sgRNA targeting efficiency is the key factor of transgenic animal generation. Multiple sgRNAs targeting one gene has been proved effective in CRISPR/Cas9-mediated gene editing (Wang et al., 2015; Yen et al., 2014). In our study, two sgRNA mixtures used together in microinjection increased the efficiency of *Scd1* knockout mice production to 50%. Of the three *Scd1* knockout mice, one had nucleotide modification at two sgRNA targeted sites. Multiple sgRNAs applied in gene editing would increase the success rate of knockout mouse production.

Although CRISPR/Cas9 technology has been used for generating gene-edited animals, it still encounters obstacles. One obstacle is mosaicism, which occurs when there are more than one genotypes in one individual, and is not desirable in most cases (Oliver et al., 2015; Yen et al., 2014). Components of the CRISPR/Cas9 system include RNA, DNA, and protein molecules, which could exist for a period of time in embryos. During different embryonic development stages, the sgRNA/Cas9 complex could induce genomic DNA double-strand break continuously, resulting in different genotypes in one individual and the formation of mosaic embryos and animals (Oliver et al., 2015). The appearance of mosaicism both in the embryos and *Scd1* knockout mice in our study demonstrated the activity of sgRNA/Cas9 after the one-cell stage. The strength of mosaicism is that a mosaic mouse with mosaic sperm or oocytes may generate different strains with different genotypes, and these are valuable genetic resources for gene function study (Mehravar et al., 2019). Whether *Scd1* mosaic knockout mice can produce different genotypes in generative cells still requires further investigation.

Delivery of a sgRNA/Cas9 system into zygotes by microinjection makes it easier for gene editing in embryos (Scott & Gruzdev, 2019). However, Cas9 that acts as an exogenous nuclease also interferes with embryo development. The negative control sgRNA that did not target any locus in the genome, together with Cas9 mRNA microinjection group, had a lower blastocyst formation rate compared with the RNase-free water injection group. Recent research has demonstrated that Cas9 expression in cell lines caused a p53-mediated DNA damage response (Haapaniemi et al., 2018) and affected cellular sensitivity to both genetic and chemical perturbations (Enache et al., 2020). More research should be conducted on the effects of the CRISPR/Cas9 system on embryo development damage.

SCD1 can catalyze MUFA synthesis by using SFAs as substrates (Ntambi et al., 2002). SCD1 protein was abundant in cumulus cells but low in oocytes, which indicated that it could protect oocytes against saturated fatty acid stress (Aardema et al., 2017). The presence of SFA would decrease the rate of oocyte maturation and blastocyst yield (VanHoeck et al., 2011). In contrast, unsaturated fatty acids could prevent the lipotoxicity induced by SFA, and the oocytes in the presence of MUFA displayed a normal development rate (Aardema et al., 2011). High palmitic acid content reduced viability and developmental ability in porcine oocytes (Shibahara et al., 2020). Additionally, knockout of fatty acid synthase, an important enzyme involved in MUFA synthesis, impaired early embryonic development (Guo et al., 2018). These results demonstrated that MUFA is crucial in embryo development, indicating the importance of SCD1 during embryogenesis. In this study, CRISPR/Cas9-mediated deficiency of SCD1 in embryos induced a significant decrease of blastocyst formation, which may have resulted from cellular toxicity caused by the accumulation of embryonic saturated fatty acids.

MUFAs and SFAs are the main components of TAG, which stores in adipose tissue and liver to provide energy for the body. When TAG is excessively accumulated in adipose tissue or hepatocytes, the possibility of obesity or non-alcoholic fatty liver disease (NAFLD) occurring is significantly increased (Engin, 2017; Frayn, Arner & Yki-Järvinen, 2006; Svegliati-Baroni et al., 2019). Leptin-deficient ob/ob mice showed lipid accumulation in their livers, and a lack of SCD1 can lead to a significantly decreased body weight (Cohen, 2002). NAFLD in neonate rats could be induced by a maternal high-fat diet through the up-regulation of SCD1 expression (Cao et al., 2020). CRISPR/Cas9-mediated SCD1 deficiency decreased lipid synthesis gene expression and TAG content in goat mammary epithelial cells (Tian et al., 2018). Overexpression of *Scd1* in primary myocytes from lean donors increased the accumulation of TAG (Hulver et al., 2005), indicating that there is an association between SCD1 expression and body fat content. Knockout of SCD1 results in the decrease of substrates during TAG synthesis, which causes the downregulation of serum TAG content and body fat. Miyazaki et al. (2007) reported that the disruption of SCD1 impaired the biosynthesis of TAG and cholesterol in liver and plasma, which supported our results. That study also demonstrated that heterozygous mice are phenotypically indistinguishable from normal mice (Miyazaki et al., 2007). However, in this study, the phenotype was different between wild type and heterozygous mice. We speculated that this may be caused by the different methods used in obtaining heterozygous mice. The off-target effects could not be predicted in the previous study, and the delivery of gene modification was unstable compared with CRISPR/Cas9-mediated gene editing. In addition, SCD1 deficiency inhibits the expression level of SREBP1 (Miyazaki et al., 2007), which is the key transcription factor in lipid and cholesterol metabolism (Yokoyama et al., 1993). Therefore, decreased TAG and cholesterol may be due to the effects of SCD1 on SREBP1. Further research should be conducted in order to explore the role of SREBP1 in *Scd1* knockout mice.

In our study, *Scd1* knockout mice were leaner compared with wild type mice, and the contents of their serum TAG and cholesterol were reduced, suggesting that SCD1 was the key regulator in the synthesis of body fat and serum lipids. Additionally, SCD1 can be used a therapeutic target of lipid metabolic disorders. However, the limitation of our study was that the obtained knockout mice were heterozygotes. In future research, mice mating will be achieved so that homozygotes can be used for further analysis of the regulatory mechanism of SCD1 in lipid metabolism.

## Conclusion

In conclusion, our study indicated that when microinjection was performed during the S phase, embryos had the highest survival rate. Using a sgRNA/Cas9 system caused damage to embryo development. Deficiency of SCD1 impaired the blastocyst formation rate and *Scd1* knockout mice showed decreased body weight, serum TAG, and cholesterol content. Our results indicated that SCD1 was the crucial regulator in embryo development and lipid synthesis. Our findings provide a method for embryo microinjection and a basis for lipid metabolic disorder therapy.

##  Supplemental Information

10.7717/peerj.13945/supp-1 Supplemental Information 1 Raw Data

10.7717/peerj.13945/supp-2 Supplemental Information 2 Original pictures of the figures including electrophoresis gel

10.7717/peerj.13945/supp-3 Supplemental Information 3 Sequence data

10.7717/peerj.13945/supp-4 Supplemental Information 4 Author Checklist - Full

## Additional Information and Declarations

### DNA Deposition

The following information was supplied regarding the deposition of DNA sequences: The sequence data is available at NCBI GeneBank BankIt: ON054180, ON054181, ON054182, ON054183, ON054184, ON054185, ON054186, ON054187.

### Data Availability

The following information was supplied regarding data availability: The raw measurements are available in the Supplementary Files.
