# Supplementary material for: Effects of CRISPR/Cas9-mediated stearoyl-Coenzyme A desaturase 1 knockout on mouse embryo development and lipid synthesis
Source: PeerJ. 2022 Sep 14;10:e13945. doi: 10.7717/peerj.13945 (PMC9482360; doi:10.7717/peerj.13945)
Supplement: Supplemental Information 3 [file peerj-10-13945-s003.docx]

**#1 sgRNA3 -2m1**

CACCTCCACGCCTGGCTTCCTTGGCTAGCTATCTCTGCGCTCTTTACCCTTTGCTGGCAGCCGATAAAAGGGGGCTGAGGAAATACTGAACACGGTCATCCCATCGCCTGCTCTACCCTTTAAAATCCCAGCCCAGGGAGATCTGTGCACAGCCAGACCGGGCTGAACACCCATCCCGAGAGTCAGGAGGGCAGGTTTCCAAGCGCAGTTCCGCCACTCGCCTACACCAACGGGCTCCGGAACCGAAGTCCACGCTCGATCTCAGCACTGGGAAAGTGAGGCGAGCAACTGACTATCATCATGCCGGCCCACACTCCAAGAGGTGAGCTTCCAGAAGCGGCCCTCGCTCCTTGCACTGGCCACGCAGCTAGGTGACTGGTCCCGGGAGGAGAAAGTTGAGAGTTCTCCGGACTACTGGGCTTTCTCGAATTCTGACGTGACCGGTTGGTTGAGAGGTGTAGATTGTAATTTGGGCAGATGAGTCCCAACTTTCGTGCCTTTAGCTTTAAAGCAGCGGTTCTCAATTTTCAAGATGCTGCGATCCTTTATTTACTACAGTTTTTCATGTTGTGGTGACCCCCTCCCCCCCAACCATAAAATGATTTCGTTGCTACTTCATAAC

**#1 sgRNA3 -9m2**

CACCTCCACGCCTGGCTTCCTTGGCTAGCTATCTCTGCGCTCTTTACCCTTTGCTGGCAGCCGATAAAAGGGGGCTGAGGAAATACTGAACACGGTCATCCCATCGCCTGCTCTACCCTTTAAAATCCCAGCCCAGGGAGATCTGTGCACAGCCAGACCGGGCTGAACACCCATCCCGAGAGTCAGGAGGGCAGGTTTCCAAGCGCAGTTCCGCCACTCGCCTACACCAACGGGCTCCGGAACCGAAGTCCACGCTCGATCTCAGCACTGGGAAAGTGAGGCGAGCAACTGACTATCATCATGCCGGCCCAAGAGGTGAGCTTCCAGAAGCGGCCCTCGCTCCTTGCACTGGCCACGCAGCTAGGTGACTGGTCCCGGGAGGAGAAAGTTGAGAGTTCTCCGGACTACTGGGCTTTCTCGAATTCTGACGTGACCGGTTGGTTGAGAGGTGTAGATTGTAATTTGGGCAGATGAGTCCCAACTTTCGTGCCTTTAGCTTTAAAGCAGCGGTTCTCAATTTTCAAGATGCTGCGATCCTTTATTTACTACAGTTTTTCATGTTGTGGTGACCCCCTCCCCCCCAACCATAAAATGATTTCGTTGCTACTTCATAAC

**#1 sgRNA3 -9m2**

CACCTCCACGCCTGGCTTCCTTGGCTAGCTATCTCTGCGCTCTTTACCCTTTGCTGGCAGCCGATAAAAGGGGGCTGAGGAAATACTGAACACGGTCATCCCATCGCCTGCTCTACCCTTTAAAATCCCAGCCCAGGGAGATCTGTGCACAGCCAGACCGGGCTGAACACCCATCCCGAGAGTCAGGAGGGCAGGTTTCCAAGCGCAGTTCCGCCACTCGCCTACACCAACGGGCTCCGGAACCGAAGTCCACGCTCGATCTCAGCACTGGGAAAGTGAGGCGAGCAACTGACTATCATCATGCCGGCCCAAGAGGTGAGCTTCCAGAAGCGGCCCTCGCTCCTTGCACTGGCCACGCAGCTAGGTGACTGGTCCCGGGAGGAGAAAGTTGAGAGTTCTCCGGACTACTGGGCTTTCTCGAATTCTGACGTGACCGGTTGGTTGAGAGGTGTAGATTGTAATTTGGGCAGATGAGTCCCAACTTTCGTGCCTTTAGCTTTAAAGCAGCGGTTCTCAATTTTCAAGATGCTGCGATCCTTTATTTACTACAGTTTTTCATGTTGTGGTGACCCCCTCCCCCCCAACCATAAAATGATTTCGTTGCTACTTCATAAC

**#2 sgRNA3 -7m1**

CACCTCCACGCCTGGCTTCCTTGGCTAGCTATCTCTGCGCTCTTTACCCTTTGCTGGCAGCCGATAAAAGGGGGCTGAGGAAATACTGAACACGGTCATCCCATCGCCTGCTCTACCCTTTAAAATCCCAGCCCAGGGAGATCTGTGCACAGCCAGACCGGGCTGAACACCCATCCCGAGAGTCAGGAGGGCAGGTTTCCAAGCGCAGTTCCGCCACTCGCCTACACCAACGGGCTCCGGAACCGAAGTCCACGCTCGATCTCAGCACTGGGAAAGTGAGGCGAGCAACTGACTATCATCATGCCGGCCCACAAGAGGTGAGCTTCCAGAAGCGGCCCTCGCTCCTTGCACTGGCCACGCAGCTAGGTGACTGGTCCCGGGAGGAGAAAGTTGAGAGTTCTCCGGACTACTGGGCTTTCTCGAATTCTGACGTGACCGGTTGGTTGAGAGGTGTAGATTGTAATTTGGGCAGATGAGTCCCAACTTTCGTGCCTTTAGCTTTAAAGCAGCGGTTCTCAATTTTCAAGATGCTGCGATCCTTTATTTACTACAGTTTTTCATGTTGTGGTGACCCCCTCCCCCCCAACCATAAAATGATTTCGTTGCTACTTCATAAC

**#2 sgRNA3 +1**

CACCTCCACGCCTGGCTTCCTTGGCTAGCTATCTCTGCGCTCTTTACCCTTTGCTGGCAGCCGATAAAAGGGGGCTGAGGAAATACTGAACACGGTCATCCCATCGCCTGCTCTACCCTTTAAAATCCCAGCCCAGGGAGATCTGTGCACAGCCAGACCGGGCTGAACACCCATCCCGAGAGTCAGGAGGGCAGGTTTCCAAGCGCAGTTCCGCCACTCGCCTACACCAACGGGCTCCGGAACCGAAGTCCACGCTCGATCTCAGCACTGGGAAAGTGAGGCGAGCAACTGACTATCATCATGCCGGCCCACAGTGCTCCAAGAGGTGAGCTTCCAGAAGCGGCCCTCGCTCCTTGCACTGGCCACGCAGCTAGGTGACTGGTCCCGGGAGGAGAAAGTTGAGAGTTCTCCGGACTACTGGGCTTTCTCGAATTCTGACGTGACCGGTTGGTTGAGAGGTGTAGATTGTAATTTGGGCAGATGAGTCCCAACTTTCGTGCCTTTAGCTTTAAAGCAGCGGTTCTCAATTTTCAAGATGCTGCGATCCTTTATTTACTACAGTTTTTCATGTTGTGGTGACCCCCTCCCCCCCAACCATAAAATGATTTCGTTGCTACTTCATAAC

**#3 sgRNA3 -7m1**

CACCTCCACGCCTGGCTTCCTTGGCTAGCTATCTCTGCGCTCTTTACCCTTTGCTGGCAGCCGATAAAAGGGGGCTGAGGAAATACTGAACACGGTCATCCCATCGCCTGCTCTACCCTTTAAAATCCCAGCCCAGGGAGATCTGTGCACAGCCAGACCGGGCTGAACACCCATCCCGAGAGTCAGGAGGGCAGGTTTCCAAGCGCAGTTCCGCCACTCGCCTACACCAACGGGCTCCGGAACCGAAGTCCACGCTCGATCTCAGCACTGGGAAAGTGAGGCGAGCAACTGACTATCATCATGCCGGCCCACAAGAGGTGAGCTTCCAGAAGCGGCCCTCGCTCCTTGCACTGGCCACGCAGCTAGGTGACTGGTCCCGGGAGGAGAAAGTTGAGAGTTCTCCGGACTACTGGGCTTTCTCGAATTCTGACGTGACCGGTTGGTTGAGAGGTGTAGATTGTAATTTGGGCAGATGAGTCCCAACTTTCGTGCCTTTAGCTTTAAAGCAGCGGTTCTCAATTTTCAAGATGCTGCGATCCTTTATTTACTACAGTTTTTCATGTTGTGGTGACCCCCTCCCCCCCAACCATAAAATGATTTCGTTGCTACTTCATAAC

**#3 sgRNA3 -1m1**

CACCTCCACGCCTGGCTTCCTTGGCTAGCTATCTCTGCGCTCTTTACCCTTTGCTGGCAGCCGATAAAAGGGGGCTGAGGAAATACTGAACACGGTCATCCCATCGCCTGCTCTACCCTTTAAAATCCCAGCCCAGGGAGATCTGTGCACAGCCAGACCGGGCTGAACACCCATCCCGAGAGTCAGGAGGGCAGGTTTCCAAGCGCAGTTCCGCCACTCGCCTACACCAACGGGCTCCGGAACCGAAGTCCACGCTCGATCTCAGCACTGGGAAAGTGAGGCGAGCAACTGACTATCATCATGCCGGCCCACAGCTCCAAGAGGTGAGCTTCCAGAAGCGGCCCTCGCTCCTTGCACTGGCCACGCAGCTAGGTGACTGGTCCCGGGAGGAGAAAGTTGAGAGTTCTCCGGACTACTGGGCTTTCTCGAATTCTGACGTGACCGGTTGGTTGAGAGGTGTAGATTGTAATTTGGGCAGATGAGTCCCAACTTTCGTGCCTTTAGCTTTAAAGCAGCGGTTCTCAATTTTCAAGATGCTGCGATCCTTTATTTACTACAGTTTTTCATGTTGTGGTGACCCCCTCCCCCCCAACCATAAAATGATTTCGTTGCTACTTCATAAC

**#3 sgRNA6 +8m1**

AATCGTAATGTAAATATCTGTTTTCCGATGGTCTTGGGCTGCCCCTCTGAAAGGGACATTTGACCCCCAAAGGGGCTGAGCGTGAGACACAGGTTAAGAACCGCTGCTTTAAAGAAAAGCCCGTCCTACCGATACTTGTATAACTTACACGGGGATCCCTTTGTGTGTTACTGAGGCTATAAATGTTTCATTCTTCTTTCCTATTTCTCTCTCTCTCTCTCTCTCACACACACAACACACAGAGAGAGACAGAGAGAGAGAGCCCCACAGATCGGCCTCCCCCGACTGAATTTTTTCTTTGCAGATCTCCAGTTCTTACACGACCACCACCACCATCACTGCACCTCCTTCCGGTTTTGCCGAAATGAACGAGAGAAGGTGAAGACGGTGCCCCTCCACCTGGAAGAAGACATCCGTCCTGAAATGAAAGAAGATATTCACGACCCCACCTATCAGGATGAGGAGGGACCCCCGCCCAAGCTGGAGTACGTCTGGAGGAACATCATTCTCATGGTCCTGCTGCACTTGGGAGGCCTGTACGGGATCATACTGGTTCCCTCCTGCAAGCTCTACACCTGCCTCTTCGGTGAGCAGCGCCCCTTGTCTTGGGCCAGTGCCTCAGATTTCTCTTTGCTCTCTAATAAGGTACCCTCTGACCCAAGGCAGTGGCTGCTCTGTTCAGATTTCCCCAGCCTTTTTTTCTGGTTCCTTGTGGGACTTAACATGAATACTGAGTCCCTGTGCAGGAGACTGGGAGGGGTCTACCTGAGGCAGAGAAGCAACTCTCTCTGAACAAGCTTTTCTGTATGAGACGCTTGCTTAAGCTGTGCATCCTGGCCCCACCTGACCCAAGAGAACCCAGCCCTACCTGAGAGGCATGGGAAATCCCGTGCTAGTCAGGGACAATTGTGGACTACTCTTCAGCCAGGGCA
